# Wide-Targeted Metabolomics Combined with Network Pharmacology Analysis Reveals Metabolite Dynamics and Optimal Harvest Time Across Developmental Stages in *Schisandra chinensis* (Turcz.) Baill.

**DOI:** 10.3390/ijms27219401

**Published:** 2026-10-22

**Authors:** Gaoyang Qu, Mingwan Sun, Shixiang Wei, Jiaming Yang, Hongbo Li, Jiushi Liu

**Affiliations:** 1 College of Horticulture, Shenyang Agricultural University, Shenyang 110866, China; qgy@syau.edu.cn (G.Q.);; 2 State Key Laboratory for Quality Ensurance and Sustainable Use of Dao-di Herbs, Institute of Medicinal Plant Development, Chinese Academy of Medical Sciences and Peking Union Medical College, Beijing 100193, China

**Keywords:** *Schisandra chinensis* (Turcz.) Baill., fruit development, harvest period, active compounds, metabolome

## Abstract

*Schisandra chinensis* (Turcz.) Baill. (*S. chinensis*) is a traditional Chinese medicinal plant whose fruits are rich in bioactive constituents, including lignans, organic acids, and flavonoids. Nevertheless, the biochemical dynamics underlying fruit development remain insufficiently characterized, limiting the effective cultivation, harvest, and utilization of *S. chinensis*. Here, we applied widely targeted metabolomics, together with a dynamic assessment of fruit growth, to profile temporal changes in major metabolites and to identify the optimal harvest window. The results show that the morphological changes in fruit mainly occurred from 70 to 110 days after fruit set; fresh fruit mass peaked at 110 days after fruit set. During the harvest period, the fruits are deep red and firm, showing no signs of softening. Meanwhile, fruit cluster weight, single fruit weight, and fruit longitudinal diameter all reached their peak values. Over the course of maturation, lignan levels declined markedly, whereas terpenoids, organic acids and flavonoids increased significantly. The most pronounced shifts were observed for schizandrin, gomisin N, shikimic acid, citric acid, gamma-terpinene and cyanidin 3-sophoroside, key marker compounds of *S. chinensis* fruit. Network pharmacology predicts that ESR1, RXRA, GATM, NCOA2, and SONEA are core targets. These findings provide a basis for determining timely harvest and for establishing quality evaluation criteria for *S. chinensis*.

## 1. Introduction

*Schisandra chinensis* (Turcz.) Baill. belongs to the genus *Schisandra* in the family *Schisandraceae* and is a woody liana. It is an important medicinal plant in China, with its mature fruit serving as the medicinal part. This plant comprises two taxonomic units: *S. chinensis* (North Wu Wei Zi) and *Schisandra sphenanthera* (South Wu Wei Zi). Compared with *S. sphenanthera*, *S. chinensis* generally offers higher and more consistent lignan and organic acid contents, cleaner wild-sourced material with richer aroma and oiliness, better drying/storage stability, and greater compliance with pharmacopoeial standards [1,2]. *S. chinensis* is mainly distributed in northeastern China, Japan, and Korea. The fruits are rich in secondary metabolites, including lignans, flavonoids, organic acids, terpenoids, and polysaccharides, that together determine product quality [3,4]. Its dried fruits are used medicinally and exhibit antitussive, hepatoprotective, anti-inflammatory, and antioxidant activities [5]. Although the fruit contains numerous active constituents, the majority of current research has concentrated on lignans [6].

*S. chinensis* produces a diverse array of bioactive compounds, among which lignans, dibenzocyclooctadiene-type secondary metabolites, are considered the principal actives, including schisandrin, schisandrins B and C, γ-schisandrin, schisantherins A and B, schisanthenol, deoxyschisandrin, and gomisins A and G [7,8,9,10]. Beyond lignans, terpenoids and flavonoids also exhibit broad biological activities, notably anti-tumor, anti-inflammatory, and antioxidant effects, and the pronounced effects on fruit aroma and color are also attributed to terpenes and flavonoid compounds [11,12]. Studies have shown that significant variations exist in the levels of volatile compounds across different colored fruits, with relative concentrations of these compounds rising as color intensity deepens. This finding indicates a correlation between fruit coloration and volatile component content [13]. Furthermore, organic acids and polysaccharides are also important metabolites accumulated in *S. chinensis* fruits. Citric acid, malic acid, and shikimic acid are the predominant organic acids, with citric acid comprising more than 60% of total acids. HPLC analyses indicate that sugars account for approximately 1.5% of the fruit, including both polysaccharides and monosaccharides; among the latter, glucose, galacturonic acid, and galactose predominate, with glucose exceeding 60% [14,15]. The accumulation of these specific compounds is responsible for the distinctive sour, bitter, sweet, pungent, and salty flavors characteristic of *S. chinensis* fruit. An untimely harvest directly results in an imbalance of these five flavors and reduction in the quality of the medicinal herb. Because the biosynthesis of these metabolites is strongly influenced by environmental factors, systematic characterization of metabolite accumulation across fruit development, coupled with elucidation of environment–biosynthesis relationships, will provide a foundation for optimized cultivation and timely harvest of *S. chinensis*.

Metabolomics provides a high-throughput approach for characterizing active constituents in medicinal plants by comprehensively profiling small molecules in biological systems [16]. Gas chromatography–mass spectrometry (GC–MS) and liquid chromatography–mass spectrometry (LC–MS) are central platforms owing to their sensitivity, resolution, and broad coverage [17]. GC–MS is well suited to measure primary metabolites [18], whereas LC–MS is used primarily for detecting secondary metabolites, including flavonoids and alkaloids [19]. Overall, metabolomics enables the identification of active ingredients, reveals the chemical basis of traditional therapeutic effects, and supports drug discovery through comprehensive chemical inventories. It is therefore poised to play an increasingly central role in the development and utilization of medicinal plant resources.

In this study, fruits of *S. chinensis* were sampled at four developmental stages after fruit set. We employed widely targeted metabolomics to profile metabolites across these stages, followed by pathway analysis to identify characteristic metabolites and quantify their temporal dynamics. These data clarify the developmental trajectories of secondary metabolites in *S. chinensis* and provide a basis for breeding and harvest timing.

## 2. Results

### 2.1. The Physiological Changes in S. chinensis Fruits During Four Maturation Processes

**Figure 1:**
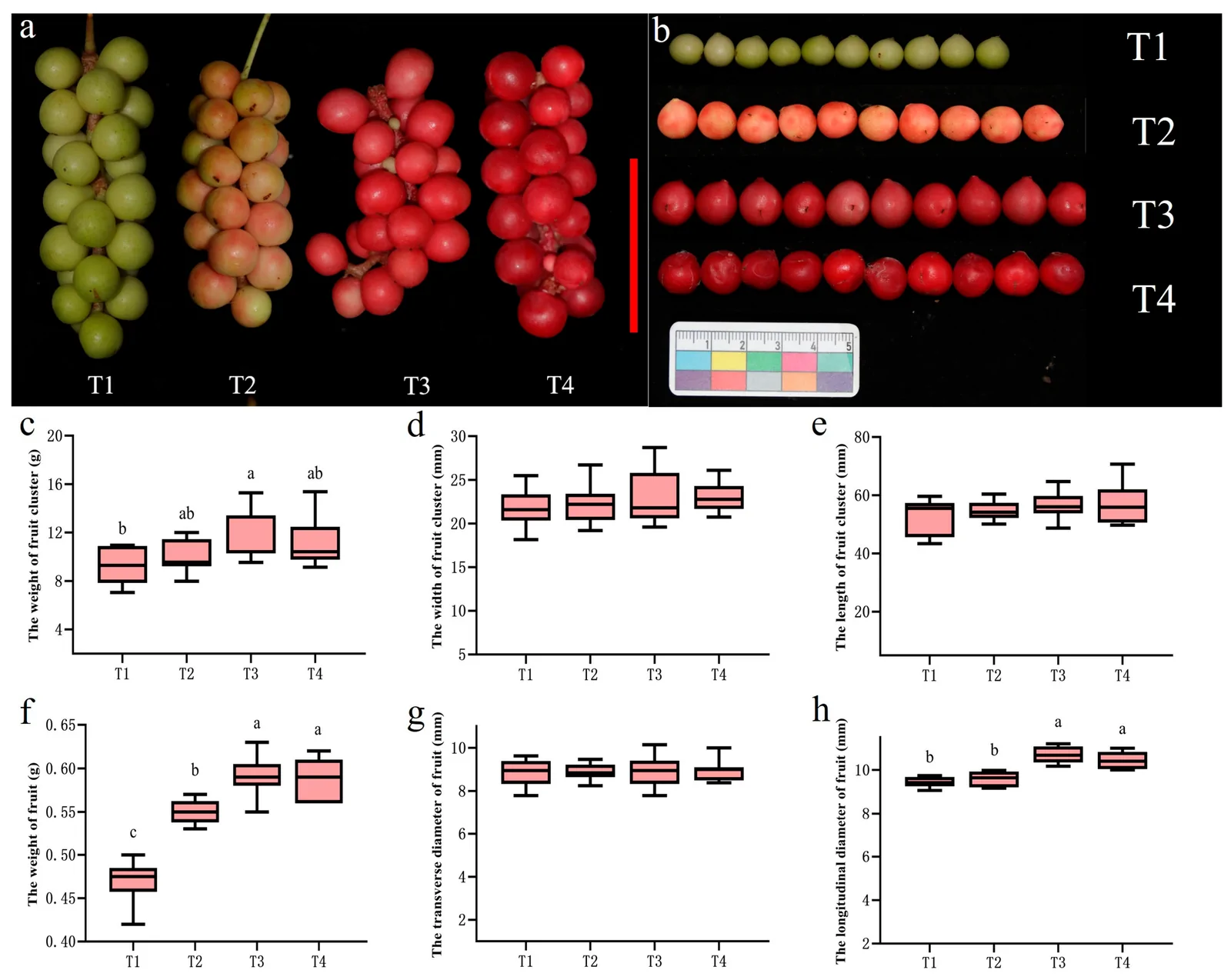
Determination of yield-related indicators during different developmental stages of *S. chinensis* fruit. (**a**) Fruit clusters at different development periods. (**b**) Fruits at different development periods. (**c**) The weight of fruit cluster. (**d**) The width of fruit cluster. (**e**) The length of fruit cluster. (**f**) The weight of fruit. (**g**) The transverse diameter of fruit. (**h**) The longitudinal diameter of fruit. Bar = 5 cm. Note: T1: fruit-setting 70 days. T2: fruit-setting 90 days. T3: fruit-setting 110 days. T4: fruit-setting 130 days. Different lowercase letters denote significant differences according to multiple comparisons (*p* < 0.05).

To characterize physiological changes across fruit development in *S. chinensis*, we sampled four post-fruit-set stages (Figure 1a,b). These developmental stages were defined based on integrated physiological criteria including fruit peel color, fruit size–weight performance, and flesh firmness, together with internal physiological indicators (anthocyanin and soluble solids contents). T1 (70 days): the fruit is relatively small, green in color, and firm in texture; T2 (90 days): the fruit expands and begins to turn partially red; T3 (110 days): the fruit quality index reaches its peak, the color turns deep red, the texture remains firm, and the contents of anthocyanins and soluble solids increase significantly; T4 (130 days): the fruit exhibits a deep red color, but the fruit texture becomes soft.

Cluster-level traits increased through development and peaked at T3. Mean single cluster fresh weights were 9.24 g, 10.01 g, 11.53 g, and 11.06 g for T1–T4, respectively (Figure 1c), with the maximum at T3 (1.82 g higher than T1). Cluster width measured 21.69 mm, 22.21 mm, 23.13 mm, and 23.00 mm across T1–T4 (Figure 1d). Cluster length increased from 52.65 mm to 57.31 mm, a net gain of 4.66 mm from T1 to T4 (Figure 1e).

At the individual fruit level, fresh weight rose from 0.47 g (T1) to 0.59 g (T3–T4), with the maximum at T3 (0.12 g higher than T1; Figure 1f). Transverse diameter remained relatively stable at 8.86, 8.89, 8.91, and 8.95 mm from T1 to T4 (Figure 1g). In contrast, longitudinal diameter increased from 9.44 to 10.70 mm by T3 and was 10.45 mm at T4 (Figure 1h). Overall, cluster weight, individual fruit weight, and fruit longitudinal diameter showed clear upward trends from T1 to T3, with slight stabilization or marginal decline by T4.

### 2.2. Multivariate Analysis of Metabolites Accumulated in S. chinensis Fruits

PCA, PLS-DA, and OPLS-DA were used to assess data quality and characterize group separation. Supervised models (PLS-DA and OPLS-DA) further enhanced group discrimination, revealing pronounced differences between stages (Figures S1 and S2). Permutation tests indicated good model stability without overfitting, with R2 and Q2 close to 1, supporting the reliability of downstream analyses. These results confirm the effectiveness of PLS-DA and OPLS-DA as predictive models for interrogating metabolic differences among samples.

**Figure 2:**
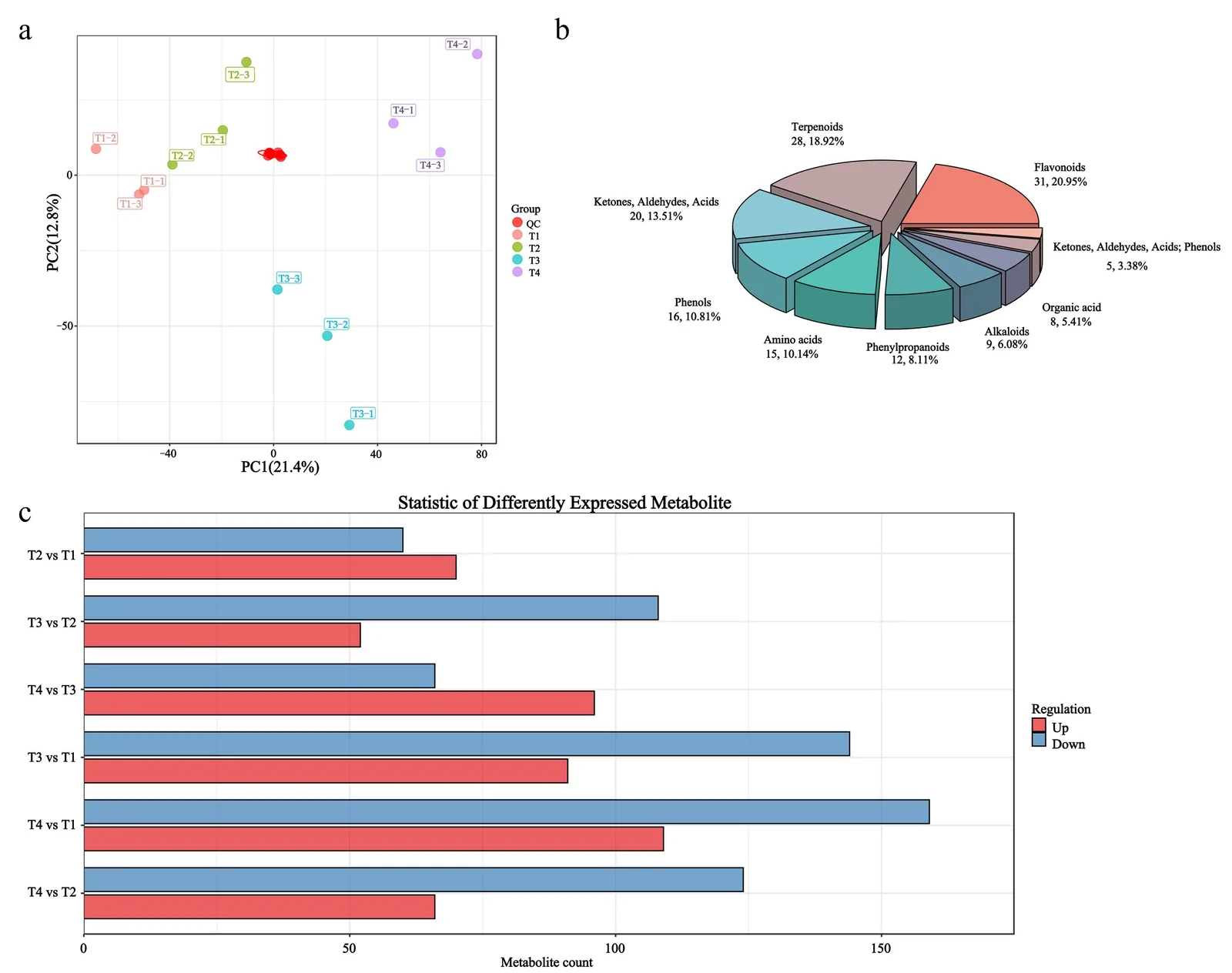
The differential metabolites during different developmental stages of *S. chinensis* fruit. (**a**) PCA analysis of different periods. Volcano plots of differential metabolites. (**b**) Distribution of differential metabolites across different classes. (**c**) The number of differential metabolites between two periods.

To comprehensively characterize metabolic changes across the growth of *S. chinensis*, we sampled four post-fruit-set stages for metabolite profiling. PCA score plots and metabolite heatmaps were used to evaluate technical reproducibility across samples, with colors denoting stages T1–T4. Biological replicates clustered tightly within each stage (Figure 2a), indicating high repeatability. The results indicate that the changes in the substances within the fruit were most significant during the T2 to T3 period.

### 2.3. Identification of Differentially Accumulated Metabolites During Four Developmental Stage

In total, 1073 metabolites were identified across the four stages by database matching, spanning flavonoids, terpenoids, phenols, phenylpropanoids, ketones, aldehydes, and organic acids (Figure 2b). Key related constituents previously reported were detected, including lignans (schizandrin, secoisolariciresinol, wuweizisu C, gomisin N), organic acids (shikimic acid, citric acid, L-malic acid, oxaloacetic acid, palmitoleic acid), sugars (arabitol, galactose 1-phosphate, D-mannose, 2-deoxy-D-glucose, trehalose), flavonoids (quercetin 3-rhamnoside-7-glucoside, procyanidin C1, quercetin 3-galactoside, cyanidin 3-sophoroside, kaempferol), terpenoids (limonene, costunolide, asiatic acid), and several phytohormones. Collectively, these metabolites shape fruit quality in *S. chinensis*.

Differentially accumulated metabolites were screened using thresholds of *p* < 0.05 and VIP > 1. Relative to T1, T2 contained 130 differentially accumulated metabolites; relative to T2, T3 contained 160 differentially accumulated metabolites; and relative to T3, T4 contained 162 differentially accumulated metabolites (Figure 2c). These results show extensive metabolic reprogramming across development, suggesting that shifts in key metabolites underpin fruit quality formation.

**Figure 3:**
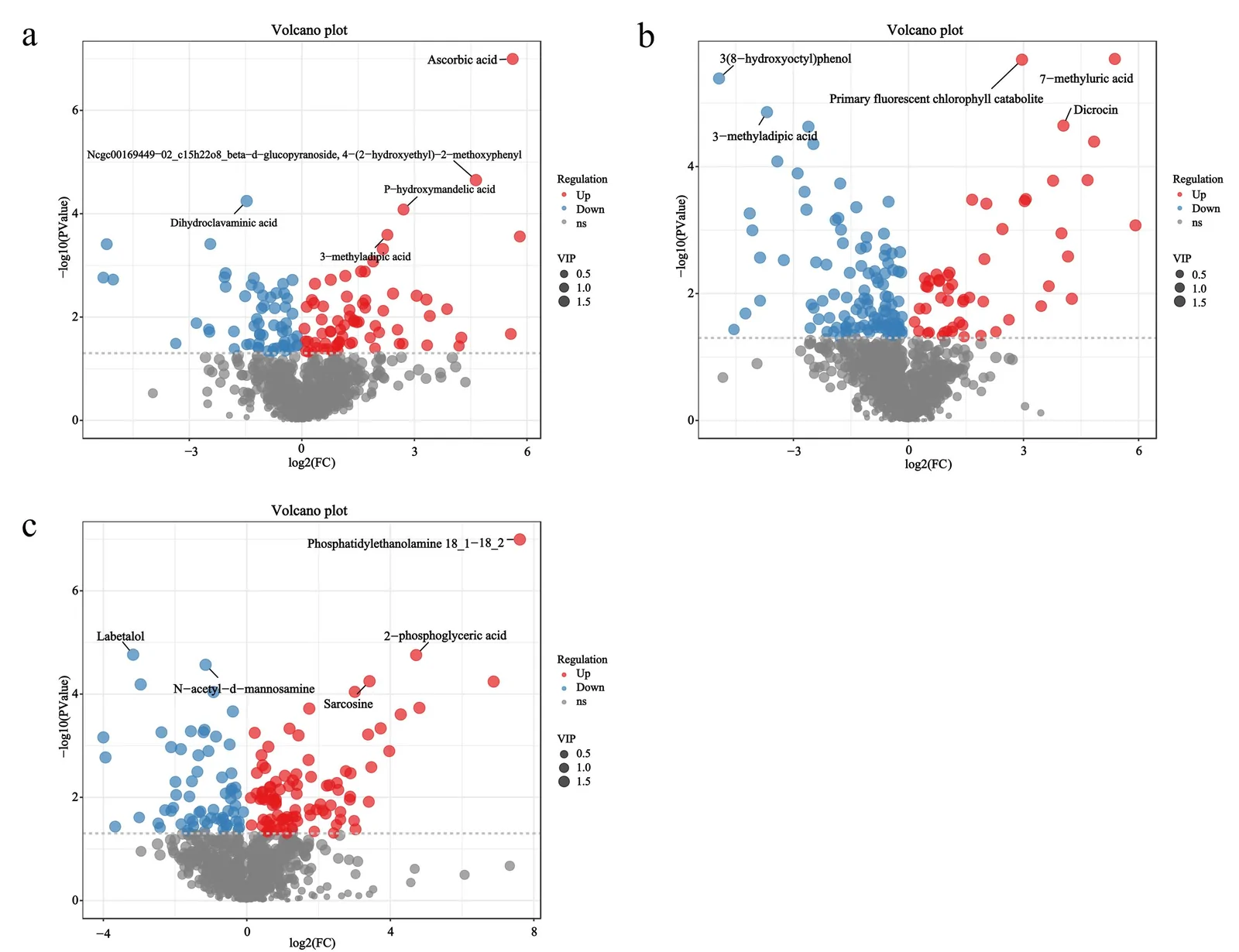
The differential metabolites during different developmental stages of *S. chinensis* fruit. (**a**) Volcano plot of differential metabolites in T2 and T1 comparison. (**b**) Volcano plot of differential metabolites in T3 and T2 comparison. (**c**) Volcano plot of differential metabolites in T4 and T3 comparison.

Among the differential metabolites found from T2 to T1, there were 70 upregulated and 60 downregulated substances (Figure 3a). Among them, the substances with the most significant changes were beta-D-glucopyranoside, 4-(2-hydroxyethyl)-2- methoxyphenyl, ascorbic acid, taxifolin, phosphatidylglyceride, phosphatidylethanolamine, phosphatidylethanolamine lyso, dihydroclavaminic acid, 3,5-dihydroxyphenylglyoxylic acid, p-hydroxymandelic acid, (r)-amphetamine (Table S1). The results revealed that taxifolin, a precursor to anthocyanin synthesis, exhibited the most significant increase during this process, indicating a crucial stage for anthocyanin accumulation. Concurrently, glycosides and ascorbic acid were synergistically upregulated, while the downregulation of phospholipids marked the fruit’s shift from developmental growth to substance accumulation. This serves as a key metabolic marker for the transition between developmental stages.

Among the differential metabolites found from T3 to T2, there were 52 upregulated and 108 downregulated substances (Figure 3b). Among them, the substances with the most significant changes were 7-methyluric acid, 3-(8-hydroxyoctyl) phenol, marini, primary fluorescent chlorophyll catabolite, dicrocin, 3-methyladipic acid, 2-[2-[(z)-pent-2-enyl]-3-[3,4,5-trihydroxy-6-(hydroxymethyl)oxan-2-yl] oxycyclopentyl] acetic acid, 6-phosphogluconic acid, 2-hydroxy-2-methylpropanal, quinic acid (Table S2). The results show that the shikimic acid intermediate product significantly increased in this stage. Jasmonic acid signal enhancement may promote the synthesis of secondary metabolites.

Among the differential metabolites found from T4 to T3, there were 96 upregulated and 66 downregulated substances (Figure 3c). Among them, the substances with the most significant changes were phosphatidylethanolamine, 9-octadecenedioic acid, (9z)-2-phosphoglyceric acid, uridine diphosphate glucose, labetalol, asiatic acid, sarcosine, 4-[2-(5-carboxy-2-hydroxy-3-methoxyphenyl) -2-oxoethylidene]-2-hydroxy-2-pentenedioate, (r)-amphetamine, lactulose (Table S3). The results show that key substances of fatty acid metabolism, glycosylation reactions, and glycolytic pathways are significantly upregulated. It is speculated that this process is a critical period for fruit development and substance accumulation.

### 2.4. Analysis of the Crucial Metabolites During Different Developmental Stages

Metabolomics revealed widespread occurrence of organic acids, terpenoids, saccharides, alkaloids, flavonoids, and phenylpropanoids in *S. chinensis* (Table 1).

With lignans constituting key components, including schizandrin, wuweizisu C, gomisin N, and secoisolariciresinol, lignan levels declined significantly during fruit ripening, particularly for schizandrin and gomisin N. Significant changes in the levels of phenylpropanoids were identified from the T1 to T4 periods. The results show that the lignan compounds schizandrin and gomisin N in the fruit significantly decreased at T2 and T3 periods. Beta-peltatin is a methyl ether derivative of α-podophyllotoxin and acts as the structural precursor for podophyllotoxin-based anti-tumor drugs. We observed a significant increase in beta-peltatin relative abundance within the fruit as it matured, suggesting that *S. chinensis* may exert a positive effect against tumors.

Beyond lignans, several other metabolites changed significantly during fruit development. Shikimic, citric, and malic acids are the predominant organic acids in *S. chinensis*. In our data, L-malic acid declined slightly throughout development, whereas citric acid decreased from T1 to T3 and then rose during ripening; neither differed significantly between T1 and T4. By contrast, shikimic acid increased continuously and was significantly higher in ripe fruit (T4) than in young fruit (T1), suggesting a key role in fruit acidity. Among saccharides, trehalose accumulated markedly from T1 to T2, and 2-deoxy-d-glucose accumulated markedly from T3 to T4, while other sugars showed no significant changes.

Terpenoids are important secondary metabolites. We identified 11 terpenoids that changed significantly in *S. chinensis* fruit across development. From T1 to T4, gibberellin a44 diacid, deoxyloganin, marrubiin, gibberellin a53, solavetivone, abscisic acid and gibberellin a51 were significantly downregulated, whereas capsidiol, gibberellin a8, sweroside, asiatic acid, dicrocin, gamma-terpinene, asperulosidic acid and gibberellin a8-catabolite were significantly upregulated. Gibberellins (GAs) and abscisic acid (ABA) are core phytohormones in plants. GAs promote cell elongation and break dormancy, whereas ABA is a classic stress-related hormone. Our study revealed that ABA relative abundance decreased significantly during fruit development, while GA relative abundance exhibited marked fluctuations, suggesting that both ABA and GA play important regulatory roles in the fruit development process. Furthermore, cis-sabinene hydrate and gamma-terpinene are the main components of the volatile oil, and mature *S. chinensis* fruits contain a large amount of volatile oil. This study found that gamma-terpinene accumulates significantly with fruit ripening, and it is speculated that it may be a key substance in the formation of volatile oil in *S. chinensis* fruits.

Flavonoids are abundant in *S. chinensis* fruit, and 11 showed significant changes across developmental stages. Levels of kaempferol, quercetin 3-rhamnoside-7-glucoside, marinin, chromen-4-one, medicarpin, and cyanidin 3-sophoroside increased markedly, whereas flavonol base + 4O, O-hex-dhex-pen, sciadopitysin, epigallocatechin, avicularin, and homoeriodictyol decreased. Because fruit color is strongly influenced by anthocyanin composition, the pronounced rise in cyanidin 3-sophoroside suggests it plays a key role in ripening and is a major contributor to *S. chinensis* fruit coloration.

### 2.5. KEGG-Based Enrichment Analysis of Differential Metabolites

**Figure 4:**
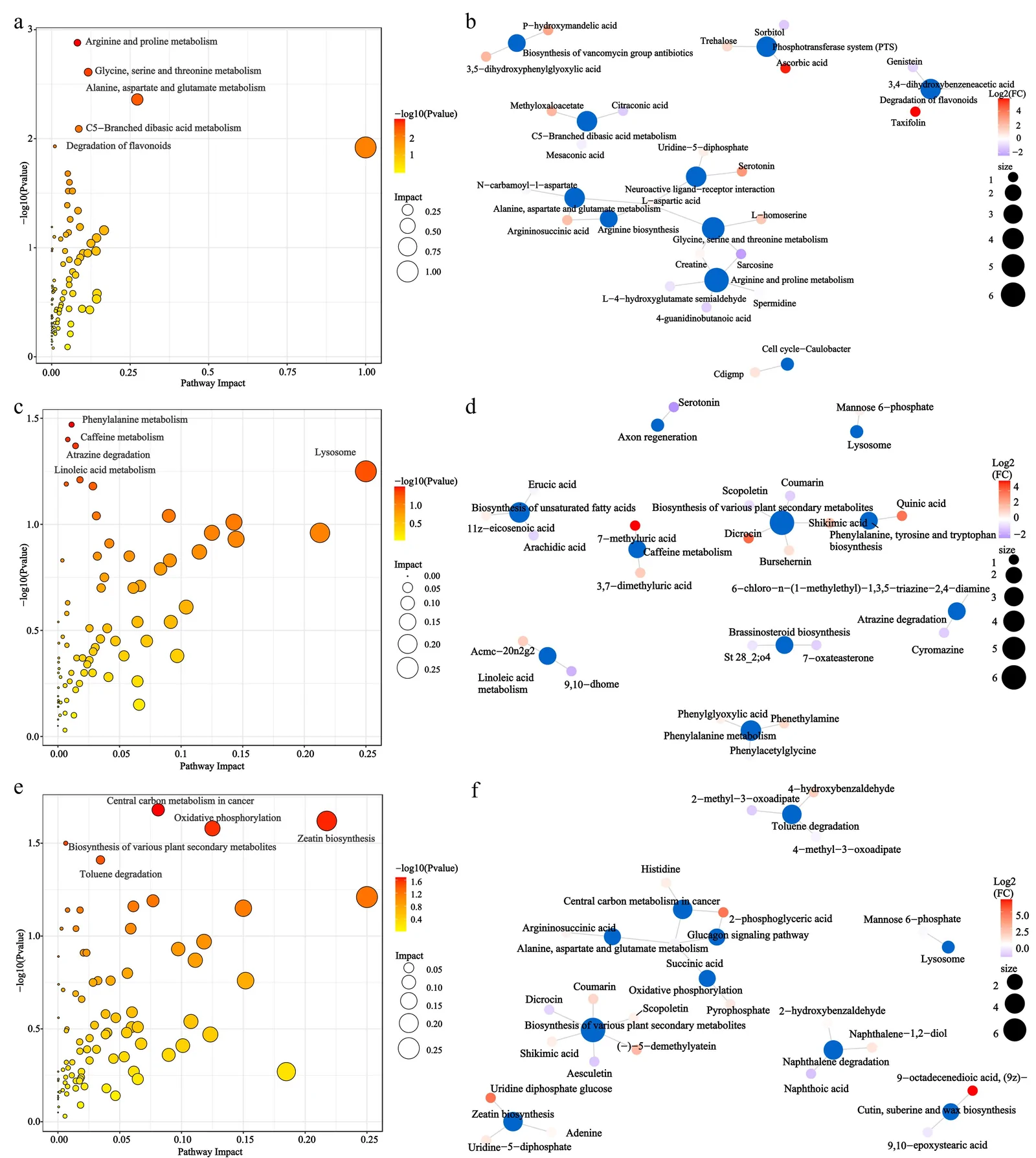
KEGG pathway enrichment analyses of different periods. (**a**) KEGG pathway enrichment analyses between T2 and T1. (**b**) KEGG pathway network between T2 and T1. (**c**) KEGG pathway enrichment analyses between T3 and T2. (**d**) KEGG pathway network between T3 and T2. (**e**) KEGG pathway enrichment analyses between T4 and T3. (**f**) KEGG pathway network between T4 and T3.

To enable intuitive comparison between groups, KEGG enrichment analysis was used to identify metabolic pathways with significant differences (*p* < 0.05). In the T2 versus T1 comparison, 12 pathways were significantly enriched: arginine and proline metabolism; glycine, serine, and threonine metabolism; alanine, aspartate, and glutamate metabolism; C5-branched dibasic acid metabolism; flavonoid degradation; cell cycle–Caulobacter; biosynthesis of vancomycin-group antibiotics; neuroactive ligand–receptor interaction; phosphotransferase system; arginine biosynthesis; pyrimidine metabolism; and cysteine and methionine metabolism (Figure 4a). Core pathway analysis indicates that differential metabolites are mainly enriched in amino acid metabolic pathways, secondary metabolite synthesis pathways, and basic substance metabolic pathways. This suggests that during this process, the fruit is primarily engaged in development and anthocyanin accumulation (Figure 4b).

In T3 versus T2, three pathways were enriched: phenylalanine metabolism, caffeine metabolism, and atrazine degradation (Figure 4c). Core pathway analysis revealed that differential metabolites were predominantly concentrated within pathways of secondary metabolite synthesis. During this process, the levels of several secondary metabolites increased significantly, encompassing phenylpropanoids and organic acids (Figure 4d).

In T4 versus T3, five pathways were enriched: central carbon metabolism in cancer, zeatin biosynthesis, oxidative phosphorylation, biosynthesis of various plant secondary metabolites, and toluene degradation (Figure 4e). Core pathway analysis showed that differential metabolites were mainly focused on core carbon metabolism and secondary metabolism pathways. Central carbon metabolism supports the basic metabolic needs of fruit development, while secondary metabolism pathways connect the synthesis of active ingredients such as phenylpropanoids and coumarins, ensuring the formation of fruit quality (Figure 4f).

### 2.6. The Changes in Components Related to the Anthocyanin and Lignan Pathways During the Mature Stage

**Figure 5:**
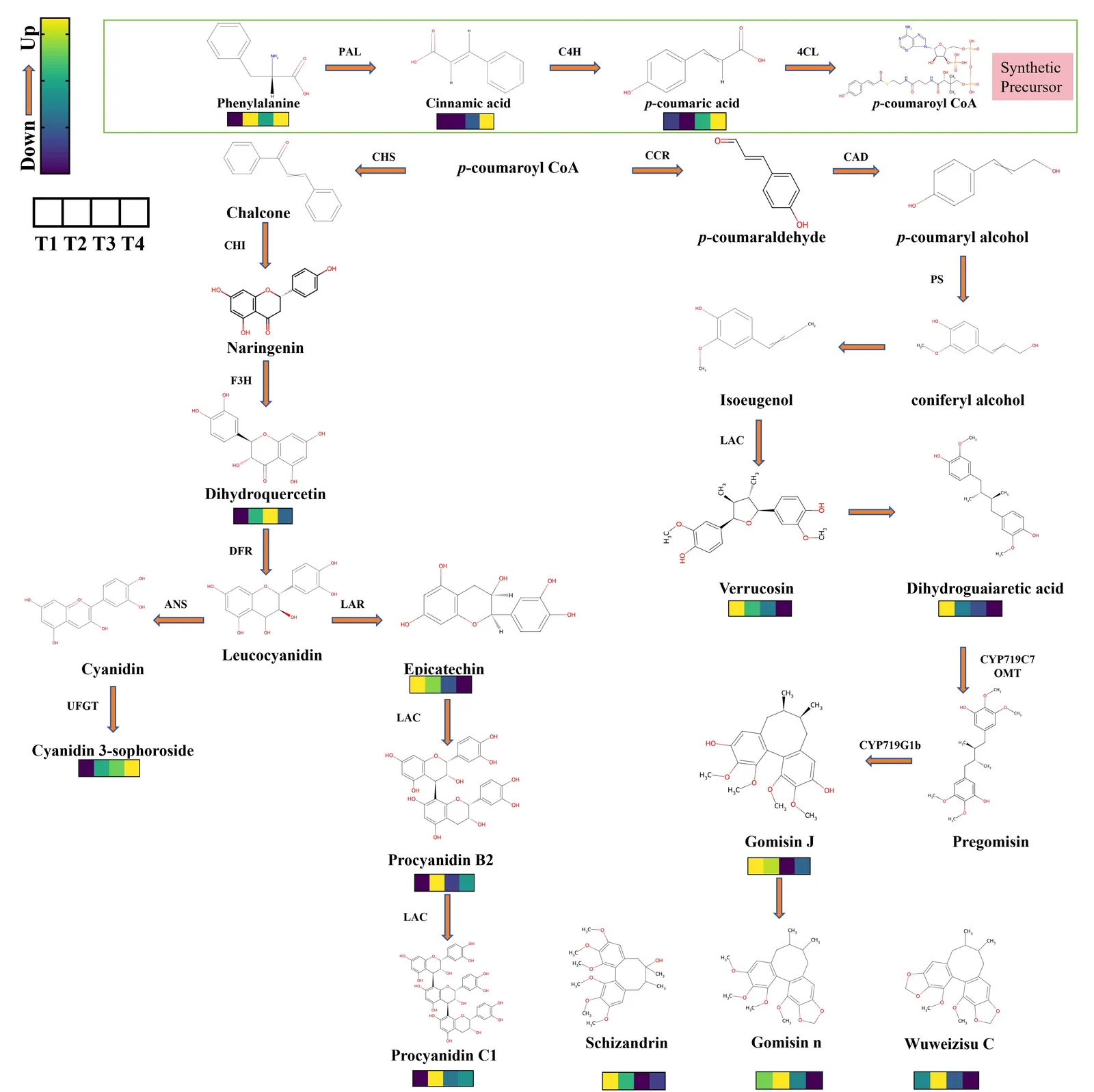
Dynamic metabolite accumulation in anthocyanin and lignan metabolic pathways during fruit development. Abbreviations: PAL, phenylalanine ammonia-lyase; C4H, cinnamate 4-hydroxylase; 4CL, 4-coumarate-CoA ligase; CHS, chalcone synthase; CHI, chalcone isomerase; F3H, flavanone 3-hydroxylase; DFR, dihydroflavonol 4-reductase; ANS, anthocyanidin synthase; UFGT, UDP-glucose flavonoid 3-O-glucosyltransferase; LAR, leucoanthocyanidin reductase; LAC, laccase; CCR, cinnamoyl-CoA reductase; CAD, cinnamyl alcohol dehydrogenase; PS, phenylpropene synthase; OMT, O-methyltransferase. Note: The color range from purple to yellow indicates that the substance relative content changes from down to up.

Metabolite profiling across four fruit developmental stages (T1–T4) revealed coordinated dynamics in anthocyanin and lignan pathways. Upstream precursors (phenylalanine, cinnamic acid and p-coumaric acid) showed progressive accumulation during maturation (Figure 5).

In the anthocyanin biosynthesis pathway, dihydroquercetin and cyanidin 3-sophoroside increase significantly with fruit ripening, with cyanidin 3-sophoroside peaking at the T4 stage. Additionally, the original proanthocyanidin pathway shows that epicatechin relative abundance declines with fruit ripening; conversely, procyanidin C1 and procyanidin B2 demonstrate an initial increase followed by a decrease in relative abundance. Within the lignin biosynthesis pathway, the levels of verrucosin, dihydroguaiaretic acid, gomisin J, schizandrin, gomisin N, and wuweizisu C all declined significantly as the fruit matured. Notably, schizandrin, as the primary active constituent, exhibited its lowest relative abundance at the T3 stage. The results show that the relative abundances of lignans and anthocyanins follow opposing trends throughout the ripening of fruit.

### 2.7. Constituent–Target–Disease Network Reveals Key Pathways and Therapeutic Associations

**Figure 6:**
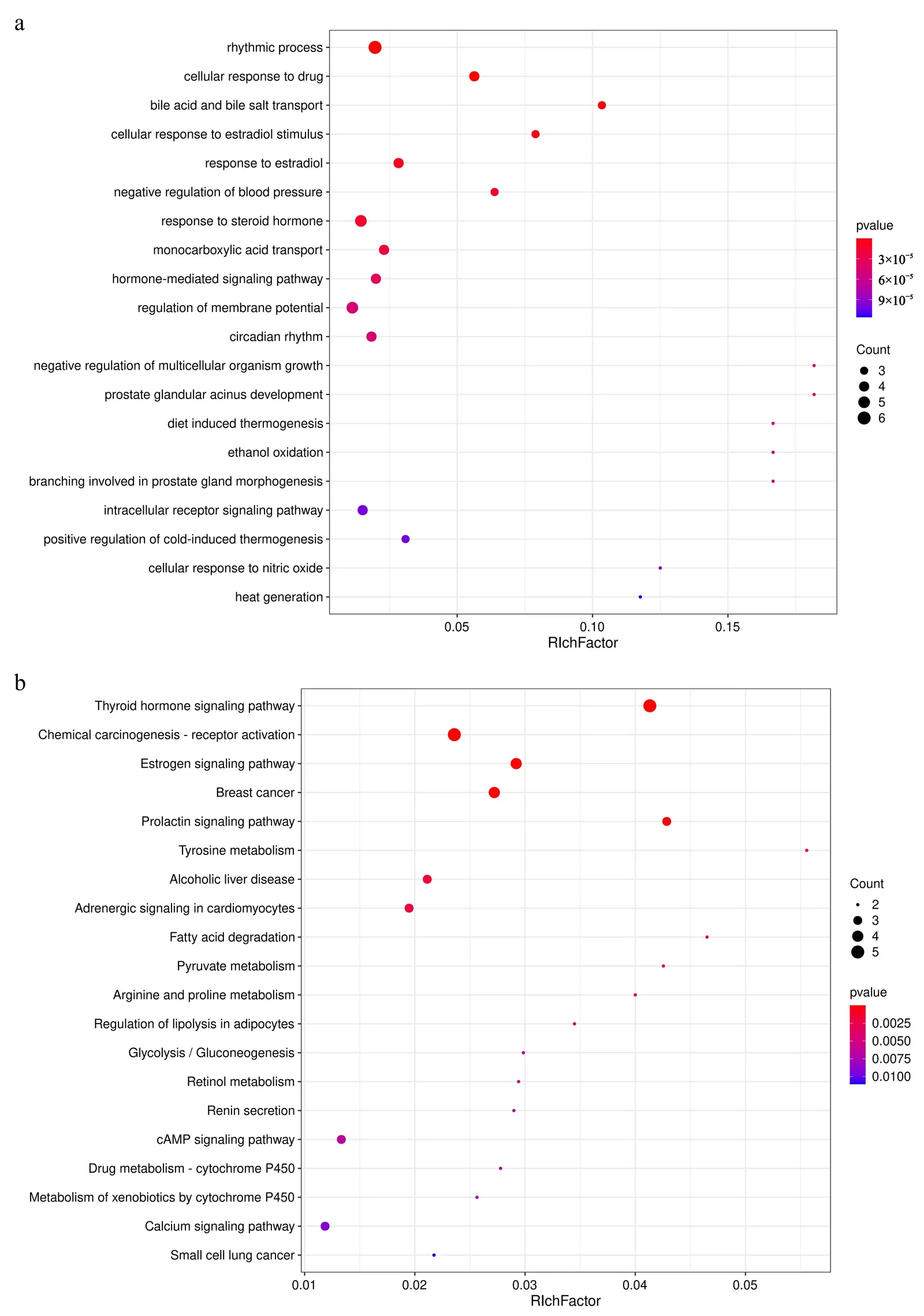
Network pharmacology analysis of active ingredients. (**a**) GO enrichment analysis of action targets. (**b**) KEGG enrichment analysis of action targets.

**Figure 7:**
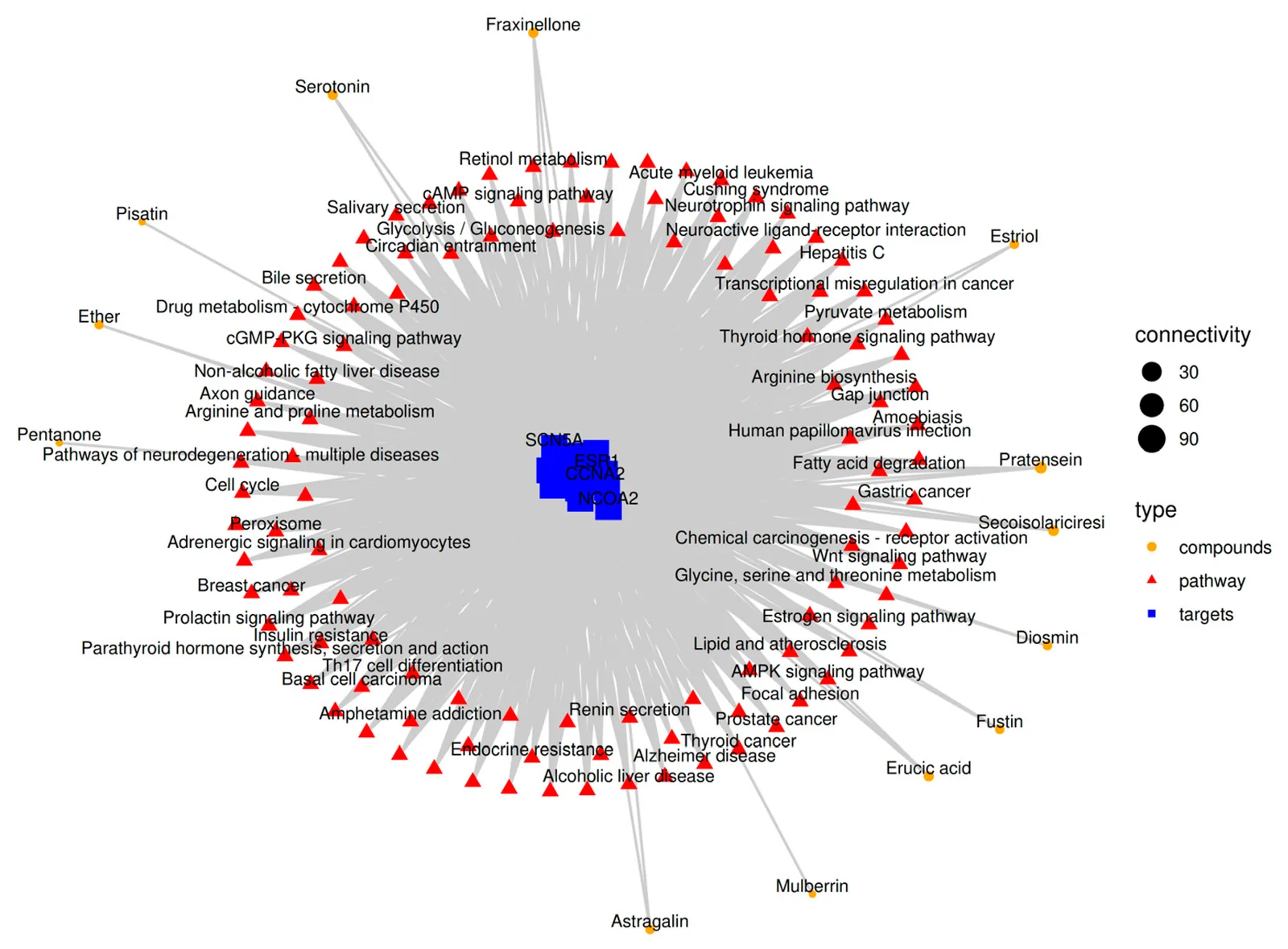
Analysis of the compound–target–pathway relationship of active ingredients.

To elucidate links between major constituents and disease pathways, we constructed an active constituent–target–disease pathway network. GO enrichment analysis indicated that 186 pathways were significantly enriched, with the most significant differences observed in rhythmic process, response to estradiol, response to steroid hormone, cellular response to drug, bile acid and bile salt transport, and negative regulation of blood pressure (Figure 6a). KEGG enrichment identified 13 key compounds connected to 15 targets and 109 pathways. Of these, 32 pathways were significantly enriched, including the thyroid hormone signaling pathway, chemical carcinogenesis–receptor activation, estrogen signaling, breast cancer, alcoholic liver disease, and tyrosine metabolism (Figure 6b). The enriched pathways mapped to 15 targets—NCOA2, SCN5A, ADRB2, GRIA2, NOS2, ESR1, GSK3B, ESR2, CCNA2, GATM, NCOA1, RXRA, ADRB1, ADH1B, and ADH1A (Figure 7). The results indicate that ESR1, RXRA, GATM, NCOA2, and SONEA are central targets within the network, as they connect numerous compounds and pathways and are likely key mediators of the drug’s pleiotropic effects. These core targets are broadly involved in multiple critical biological signaling pathways, particularly the cAMP and Wnt signaling pathways, glycolysis/gluconeogenesis, and various cancer-related pathways.

Database annotations also linked these compounds to diseases such as alcoholic liver disease, thyroid cancer, breast cancer, Alzheimer’s disease, and dermatologic disorders (Figure S3). The results indicate that mulberrin, fraxinellone, astragalin, secoisolariciresinol, serotonin, and estriol are the important substances. Together, these findings define an effective constituent–target–disease network and suggest new avenues for biomedical investigation.

## 3. Discussion

*S. chinensis* is a traditional medicinal plant whose fruits, known as “Wuweizi,” are reputed to embody five tastes, sour, sweet, bitter, pungent, and salty. It is commonly used for kidney tonification, sedation, hepatoprotection, and support of nervous and digestive functions [8]. Lignans are widely regarded as the principal bioactive constituents, and most research to date has focused on this class, with other metabolites receiving comparatively less attention. Using chemical databases and the Progenesis QI platform, Liu et al. (2022) identified 332 compounds across different tissues, including 76 markers, primarily lignans, triterpenoids, and flavonoids [20]. HPLC and HS-GC-IMS analyses across harvesting periods showed that lignan levels peaked in July and declined during ripening, whereas organic acids increased; 67 volatile flavor compounds were also detected, dominated by terpenoids [21]. In this study, we applied widely targeted metabolomics to profile metabolites in *S. chinensis* fruits at multiple developmental stages. We identified 1073 compounds, including 12 phenylpropanoids, 31 flavonoids, 28 terpenoids, 16 phenols, 15 amino acids, plus additional sugars and organic acids, representing, based on our literature survey, one of the most extensive metabolite catalogs reported for *S. chinensis* fruit. Consistent with previous work, lignans were significantly downregulated during ripening, while organic acids and flavonoids increased. These findings provide a foundation for optimized development, utilization, harvest timing, and processing of *S. chinensis* fruit.

Lignans are key secondary metabolites whose biosynthesis is shaped by environmental cues and hormone signaling through complex regulatory networks. In *Isatis indigotica*, the AP2/ERF factor *Ii049* binds promoters of *IiPAL* and *IiCCR*, key genes in the lignin/lignan pathway, to activate their expression by promoting salicylic acid synthesis, thereby enhancing lignan accumulation [22]. Polyamine signaling also stimulates lignan production by upregulating PAL and PLR via activation of diamine oxidase and NADPH oxidase [23]. In *tetraploid I. indigotica*, *WRKY34* enhances stress tolerance and activates the rate-limiting enzyme gene *Ii4CL3*, redirecting carbon flux into the phenylpropanoid pathway to promote lignan accumulation and biomass [24]. In flax hairy roots, hydrogen sulfide signaling boosts podophyllotoxin and other lignans by fine-tuning the antioxidant system and phenylpropanoid branch-point balance while suppressing certain flavonoids [25]. Hormone–stress crosstalk further refines regulation: in flax, ABA controls lignan biosynthesis by regulating the expression of PLR via Ca2+ signaling [26]. In our dataset, lignans declined progressively with ripening, coincident with a significant decrease in ABA and an increase in multiple GAs, suggesting that ABA and GA may modulate lignan accumulation in fruit. Environmental stresses further modulate lignan biosynthesis via transcriptional control. In developing *S. chinensis* fruit, phenylpropanoid and ABA signaling integrate to form a transcriptional network that precisely regulates the expression of lignan-biosynthetic genes *IGS1* and *DIR* [27]. Together, these observations offer mechanistic hypotheses for regulating lignan accumulation in *S. chinensis*.

Organic acids and anthocyanins are major determinants of fruit quality [28]. Anthocyanins impart the red coloration of *S. chinensis* fruit and contribute to its antioxidant capacity. Full-length transcriptomics combined with targeted metabolomics has delineated the anthocyanin pathway in this species and identified cyanidin 3-O-xyl-rutinoside as a predominant anthocyanin that accumulates rapidly from approximately 80 days after flowering [29]. Our data indicate that cyanidin 3-sophoroside may be the principal pigment driving color change from green (T1) to red (T4), as its levels increased continuously and significantly across development. Citric acid is the major organic acid in *S. chinensis* fruit, accounting for roughly 18% of total organic acids and conferring pronounced acidity [30]. Unlike some prior reports, we observed substantial developmental shifts in organic acids, particularly shikimic and citric acids, with shikimic acid showing the largest increase. We also found coordinated increases in organic acids and anthocyanins, suggesting a functional linkage. Organic acids can stabilize anthocyanins by lowering pH, thereby enhancing antioxidant activity. UPLC-Q-TOF/MS profiling confirmed co-occurrence of organic acids and anthocyanins and proposed synergistic contributions to pharmacological activity [31]. HS-GC-IMS analysis revealed strong correlations between citric acid and anthocyanin-related metabolites [32]. These insights advance understanding of anthocyanin accumulation in *S. chinensis* and support strategies to co-enhance organic acids and anthocyanins during cultivation or processing.

Harvest timing critically affects the yield and quality of medicinal plants [33]. Active constituents, biomass allocation, and medicinal value vary across growth stages. For example, in *Salvia miltiorrhiza*, tanshinone IIA, cryptotanshinone, and salvianolic acid B are higher at 180 days after transplanting than at 60 days, with some constituents nearing peak at 120 days [34]. In *Chrysanthemum*, luteolin, apigenin, and 3,5-O-dicaffeoylquinic acid peak at early and full flowering and decline at bud and senescence stages [35]. In *Perilla frutescens* leaves, terpenes, phenolic acids, and flavonoids are most abundant pre-flowering and subsequently decrease [36]. In *S. chinensis*, we detected pronounced developmental shifts in major constituents. Schizandrin, wuweizisu C, gomisin N, and alkaloids peaked at T1 and then declined sharply, whereas organic acids, anthocyanins, and polysaccharides increased with maturity. Accordingly, fruits harvested at 70 days after fruit set are recommended for lignan extraction, while those harvested at 130 days are preferable for organic acids and anthocyanins.

Delaying harvest generally increases biomass but can decrease the relative abundance of active constituents per unit mass. For example, in *Lactuca indica*, the chicoric acid content in leaves and stems peaks at the bud stage, whereas seed content is the highest at the fruiting stage [37]. Similarly, in *Fritillaria cirrhosa*, the dry weight of an individual bulb reaches its maximum at the wilting stage, but total alkaloid content is the highest at full flowering stage [38]. In our study, fruit morphology changed markedly throughout development, with fruits at stage T3 attaining the largest size and maximum yield. However, over the subsequent 20 days, yield declined slightly, while the relative abundance of organic acids and other metabolites increased substantially. Taken together, these results indicate that the optimal harvest time for *S. chinensis* fruit is 110 days (T3 stage) after fruit set.

## 4. Materials and Methods

### 4.1. Plant Materials and Sample Preparation

The plant material used in this study consisted of a self-pollinated inbred line of northern *S. chinensis*; all individuals were grown at the Chinese herbal cultivation base of Shenyang Agricultural University. All sampled individuals were six-year-old individuals exhibiting uniform growth. Fifteen individuals of comparable vigor were chosen, and each group of five individuals served as a single replicate. Five fruit clusters were harvested per plant, and the collected fruits were combined for subsequent mixed-sample analysis. Fruits were collected at four developmental stages, 70, 90, 110, and 130 days after fruit set (T1, T2, T3, and T4), for metabolite extraction.

After whole fruits were ground, 100 mg of the obtained powder was weighed into a 2 mL centrifuge tube, and 600 µL MeOH supplemented with 4 ppm 2-amino-3-(2-chloro-phenyl)-propionic acid was added. The mixture was vortexed for 30 s, steel balls were introduced, and tissue homogenization was conducted at 55 Hz for 60 s. Room-temperature sonication was applied for 15 min. Following centrifugation at 12,000 rpm and 4 °C for 10 min, the supernatant was filtered via a 0.22 μm membrane and transferred into an autosampler vial for LC-MS analysis.

### 4.2. LC-MS Conditions

Liquid chromatography was performed on a Vanquish UHPLC system (Thermo Fisher Scientific, Waltham, MA, USA) using an ACQUITY UPLC HSS T3 column (2.1 × 100 mm, 1.8 µm; Waters, Milford, MA, USA) maintained at 40 °C. The flow rate was 0.3 mL/min and the injection volume was 2 µL. For LC-ESI(+)-MS, the mobile phases were 0.1% formic acid in water (A2, *v*/*v*) and 0.1% formic acid in acetonitrile (B2, *v*/*v*). The gradient was as follows: 0–1 min, 8% B2; 1–8 min, 8–98% B2; 8–10 min, 98% B2; 10–10.1 min, 8–98% B2; 10.1–12 min, 8% B2. For LC-ESI(-)-MS, the mobile phases were 5 mM ammonium formate in water (A3) and acetonitrile (B3). The gradient was: 0–1 min, 8% B3; 1–8 min, 8–98% B3; 8–10 min, 98% B3; 10–10.1 min, 8–98% B3; 10.1–12 min, 8% B3.

Mass spectrometric detection was carried out on a Q Exactive mass spectrometer (Thermo Fisher Scientific, Waltham, MA, USA) with an ESI source in Full MS-ddMS2 (data-dependent MS/MS) mode. Key parameters were as follows: sheath gas, 40 arb; auxiliary gas, 10 arb; spray voltage, 3.50 kV (ESI+) and −2.50 kV (ESI-); capillary temperature, 325 °C; MS1 range, m/z 100–1000; MS1 resolving power, 70,000 FWHM; data-dependent MS2 scans per cycle, 10; MS2 resolving power, 17,500 FWHM; normalized collision energy, 30 eV; dynamic exclusion, automatic.

### 4.3. Metabolome Data Processing

Raw data were converted to mzXML format and processed in R using XCMS v3.12.0 for feature detection, retention-time correction, and alignment [39]. Key parameters were as follows: ppm = 15, peakwidth = c (5, 30), mzdiff = 0.01, and method = centWave. Batch effects were corrected using QC samples, and metabolites with RSD > 30% in QC samples were removed prior to downstream analysis.

Metabolite annotation was based on accurate mass and MS/MS spectra matched against HMDB v5.0, LipidMaps, KEGG, ChEBI, and an in-house library from Panomix Biomedical Tech Co., Ltd. (Suzhou, China) [40]. Molecular weights were inferred from parent-ion m/z in MS data; molecular formulas were predicted using mass accuracy (ppm) and adduct information and then matched to databases for MS-level identification. MS/MS spectra from the quantitative dataset were subsequently compared with database fragment ions and related metadata to achieve MS/MS-level confirmation.

### 4.4. Identification of Differentially Accumulated Metabolites

Unsupervised and supervised multivariate models (PCA, PLS-DA, and OPLS-DA) were applied using the R package ropls v1.22.0 to discriminate sample groups [41]. Between-group statistical tests provided *p* values, and *p* values were derived from Student’s *t*-tests. Together with VIP scores from OPLS-DA and fold change (FC), these were used to screen candidate biomarkers. By default, metabolites with *p* < 0.05 and VIP > 1 were considered to exhibit significantly differential accumulation.

### 4.5. Pathway Enrichment Analysis of Differentially Accumulated Metabolites

Differentially accumulated metabolites were analyzed in MetaboAnalyst [42], integrating pathway enrichment with pathway topology analysis. Identified metabolites were mapped to KEGG pathways to facilitate biological interpretation at the systems level, and metabolites and their associated pathways were visualized using KEGG Mapper.

### 4.6. Network Pharmacology Analyses

Targets of active compounds in traditional Chinese medicine (TCM) and disease-associated targets were retrieved from specialized TCM and disease databases. Network pharmacology analysis was used to investigate potential connections between key differential metabolites and disease-associated targets. Only the key differential metabolites identified from metabolomic screening were used for target discovery. Experimentally verified metabolite–protein interactions were sourced from STITCH (v5.0), HMDB (v5.0), and CTD databases; putative targets were predicted with SwissTargetPrediction, keeping those with a prediction probability exceeding 0.3. The UniProt database was used to map protein identifiers to human gene symbols. Disease-related genes were obtained from GeneCards (v5.16) and DisGeNET (v7.0), and Venny2.1 was employed to determine the overlap between metabolite targets and disease genes. These overlapping genes were submitted to STRING (v11.5, Homo sapiens, confidence score > 0.7) to generate a protein–protein interaction (PPI) network, which was visualized via Cytoscape v3.9.1 [43].

### 4.7. Statistical Analysis

Data analyses were performed on the experimental data using Excel 2022 and SPSS 26.0. GraphPad Prism 10.6 was used to create the figures.

## 5. Conclusions

This study employed widely targeted metabolomics to characterize metabolite variations in *S. chinensis* fruits across different developmental stages. The results revealed that lignan relative abundance, the primary bioactive constituent, decreased significantly with fruit maturation, while organic acid and anthocyanin levels increased substantially. Among these metabolites, schizandrin, gomisin N, shikimic acid, citric acid, gamma-terpinene and cyanidin 3-sophoroside exhibited the most pronounced changes and may serve as marker compounds for monitoring fruit development. Additionally, fruit morphology changed markedly during maturation, with maximum yield occurring at stage T3. Network pharmacology analysis found that ESR1, RXRA, GATM, NCOA2, and SONEA are core targets. These findings identify key marker metabolites throughout the fruit developmental process, thereby establishing a theoretical framework for determining the optimal harvest time of *S. chinensis* fruits.

## Figures and Tables

**Table 1:** Identification of key differential metabolites during the developmental stages.

Class	Compounds	Formula	CAS	log_2_FC (T2VST1)	log_2_FC (T3VST2)	log_2_FC (T4VST3)	log_2_FC (T4VST1)	Prediction Function or Associated Diseases
Organic acid	Alpha-ketoisovaleric acid	C_5_H_8_O_3_	759-05-7	0.76	3.66	0.77	5.2	Colorectal cancer; maple syrup urine disease
	2-ketobutyric acid	C_4_H_6_O_3_	600-18-0	1.21	0.28	0	1.49	Eosinophilic esophagitis
	Succinic acid semialdehyde	C_4_H_6_O_3_	692-29-5	1.23	−0.66	1.13	1.71	Surfactant
	Shikimic acid	C_7_H_10_O_5_	138-59-0	1.03	1.66	0.65	3.34	Prostate cancer
	Citric acid	C_6_H_8_O_7_	77-92-9	−0.41	−0.36	1.2	0.43	Hyperoxalemia; colorectal cancer; pregnancy
	L-malic acid	C_4_H_6_O_5_	97-67-6	−0.14	−0.13	−0.82	−1.1	Colorectal cancer; schizophrenia; anoxia
Terpenoids	Picrotoxinin	C_15_H_16_O_6_	17617-45-7	−0.32	−0.21	−0.47	−1	None
	Gibberellin a44 diacid	C_20_H_28_O_6_	88373-65-3	−0.29	−0.86	−1.16	−2.3	Plant hormone
	Capsidiol	C_15_H_24_O_2_	37208-05-2	−0.16	−1.35	0.49	−1.01	Fungicide; pesticide; phytoalexin
	Deoxyloganin	C_17_H_26_O_9_	26660-57-1	−0.34	0.69	−1.38	−1.03	None
	Gibberellin a8	C_19_H_24_O_7_	7044-72-6	0.59	−1.64	2.61	1.56	Plant hormone
	Marrubiin	C_20_H_28_O_4_	465-92-9	−0.12	−1.75	0.86	−1.01	None
	Sweroside	C_16_H_22_O_9_	14215-86-2	−0.12	−0.06	1.19	1.01	None
	Gibberellin a53	C_20_H_28_O_5_	51576-08-0	0.87	−0.85	−2.08	−2.06	Plant hormone
	Cis-sabinene hydrate	C_10_H_18_O	15537-55-0	−0.6	0.37	−0.84	−1.07	None
	Solavetivone	C_15_H_22_O	54878-25-0	0.3	−1.26	0.15	−0.81	Fungicide; pesticide; surfactant; phytoalexin
	Abscisic acid	C_15_H_20_O_4_	21293-29-8	−0.37	−1	−1.05	−2.42	Plant hormone
	Gibberellin a51	C_19_H_24_O_5_	56978-14-4	−0.14	−2.59	1.16	−1.57	Plant hormone
	Asiatic acid	C_30_H_48_O_5_	464-92-6	3.71	−3.95	4.29	4.06	Antioxidant; anti-inflammatory
	Dicrocin	C_32_H_44_O_14_	57710-64-2	5.57	4.04	−1.29	8.32	None
	Gamma-terpinene	C_10_H_16_	99-85-4	0.72	1.1	0.3	2.12	Nonalcoholic fatty liver disease
	Asperulosidic	C_18_H_24_O_12_	25368-11-0	1.04	−0.59	1.26	1.7	None
	Gibberellin a8-catabolite	C_19_H_22_O_7_	78791-79-4	0.02	−1.17	1.53	0.37	Plant hormone
Saccharides	2-deoxy-d-glucose	C_6_H_12_O_5_	154-17-6	−0.17	−0.82	1.15	0.17	None
	Trehalose	C_12_H_22_O_11_	99-20-7	1.13	−0.05	0.12	1.19	Pharmaceutical
Flavonoids	Kaempferol-3-rutinoside	C_27_H_30_O_15_	17650-84-9	0.51	−4.14	3.97	0.34	None
	Tricetin	C_15_H_10_O_7_	520-31-0	1.34	−0.52	−0.15	0.67	Anti-inflammatory; pharmaceutical
	Kaempferol	C_15_H_10_O_6_	520-18-3	0.51	−4.14	0.56	1.6	Colorectal cancer
	Quercetin 3-rhamnoside-7-glucoside	C_27_H_30_O_16_	17306-45-5	1.71	2.28	−1.97	2.02	Nutrient
	Apigenin 7-o-beta-d-rutinoside	C_27_H_30_O_14_	17306-46-6	0.88	−0.48	−1.18	−0.78	None
	Theaflavin digallate	C_43_H_32_O_20_	30462-35-2	−0.18	−0.34	−0.74	−1.27	Anti-methicillin-resistant Staphylococcus aureus
	Flavonol base + 4o, o-hex-dhex-pen	C_32_H_38_O_20_	129235-39-8	−0.74	−0.36	−2.49	−3.58	Nutrient
	Sciadopitysin	C_33_H_24_O_10_	521-34-6	−0.16	−0.71	−1.74	−2.62	Nutrient; anti-tumor
	Cosmosiin	C_21_H_20_O_10_	578-74-5	0.42	1.27	−0.67	1.02	Nutrient
	Procyanidin c1	C_45_H_38_O_18_	37064-30-5	1.96	−0.87	0.22	1.31	Antioxidant; enzyme inhibitor
	(+)-epicatechin	C_15_H_14_O_6_	35323-91-2	1.44	−1.17	−0.3	−0.04	Nutrient
	Quercetin 3-galactoside	C_21_H_20_O_12_	482-36-0	−0.24	0.35	−1.6	−1.49	None
	3-o-methylviolanone	C_18_H_18_O_6_	56973-42-3	0.81	0.57	2.23	3.61	None
	Marini	C_25_H_28_O_6_	97938-30-2	4.1	4.84	−0.59	8.35	None
	Epigallocatechin	C_15_H_14_O_7_	970-74-1	−0.83	0.01	−1.53	−2.35	Anticancer agent; nutrient; antioxidant
	Avicularin	C_20_H_18_O_11_	572-30-5	0.45	−2.56	−3.67	−5.77	Antibiotic; diuretic; pesticide
	Medicocarpin	C_22_H_24_O_9_	52766-70-8	3.87	−0.92	2.05	5	Nutrient
	Homoeriodictyol	C_16_H_14_O_6_	446-71-9	−0.51	−0.58	−1.38	−2.47	None
	Cyanidin 3-sophoroside	[C_27_H_31_O_16_]^+^	No CAS	2.01	0.25	0.29	2.56	Nutrient; antioxidant
Phenylpropanoids	Schizandrin	C_24_H_32_O_7_	7432-28-2	−0.38	−1.24	0.47	−1.14	Liver protection; antioxidant; anti-inflammatory; anti-tumor
	Chavicol	C_9_H_10_O	501-92-8	1.11	−1.31	1.21	1.02	Fungicide; nematicide; pesticide
	Isofraxidin	C_11_H_10_O_5_	486-21-5	−0.74	−0.59	0.26	−1.06	Anti leukemic; antimalarial; choleretic; cancer preventive
	Beta-peltatin	C_22_H_22_O_8_	518-29-6	0.56	−0.34	1.32	1.54	None
	Paulownin	C_20_H_18_O_7_	13040-46-5	−1.27	−0.62	−2.47	−4.35	None
	Syringin	C_17_H_24_O_9_	118-34-3	−0.31	−0.74	0	−1.05	None
	3,4-dihydro-2h-1-benzopyran-2-one	C_9_H_8_O_2_	119-84-6	−0.1	−0.88	−1.79	−2.77	None
	Gomisin N	C_23_H_28_O_6_	69176-52-9	0.23	−0.73	−1.06	−1.56	None

Note: Class: primary category of compounds; compounds: metabolite compounds; formula: compound molecular formula; CAS: compound CAS number; log_2_FC (A vs. B): represents the logarithmic value of the fold change in the relative abundance of a substance in A relative to B. A positive value indicates that the relative abundance of the substance is upregulated in A, while a negative value indicates that the relative abundance of the substance is downregulated in A. Prediction functions or associated diseases were predicted using the HMDB v5.0 database.

## Data Availability

The original contributions presented in this study are included in the article/Supplementary Materials. Further inquiries can be directed to the corresponding authors.

## Supplementary Materials

The following supporting information can be downloaded at https://www.mdpi.com/article/10.3390/ijms27219401/s1.

## References

[1] Liu H., Lai H., Jia X., Liu J., Zhang Z., Qi Y., Zhang J., Song J., Wu C., Zhang B. (2013). Comprehensive chemical analysis of *Schisandra chinensis* by HPLC-DAD-MS combined with chemometrics. Phytomed. Int. J. Phyther. Phytopharm..

[2] Zhang L., Tang H., Zheng J., Yu Q., Wang H., Li D., Zhang Z., Tang Z. (2026). Comparative metabolomic evaluation of *Schisandraceae* leaves as novel food ingredients: Nutritional quality and biofunctional components across genera. Food Res. Int..

[3] Yang K., Qiu J., Huang Z., Yu Z., Wang W., Hu H., You Y. (2022). A comprehensive review of ethnopharmacology, phytochemistry, pharmacology, and pharmacokinetics of *Schisandra chinensis* (Turcz.) Baill. and *Schisandra sphenanthera* Rehd. et Wils. J. Ethnopharmacol..

[4] Jia M., Zhou L., Lou Y., Yang X., Zhao H., Ouyang X., Huang Y. (2023). An analysis of the nutritional effects of *Schisandra chinensis* components based on mass spectrometry technology. Front. Nutr..

[5] Olas B. (2023). Cardioprotective potential of berries of *Schisandra chinensis* Turcz. (Baill.), their components and food products. Nutrients.

[6] Zhang F., Zhai J., Weng N., Gao J., Yin J., Chen W. (2022). A comprehensive review of the main lignan components of *Schisandra chinensis* (North Wu Wei Zi) and *Schisandra sphenanthera* (South Wu Wei Zi) and the lignan-induced drug-drug interactions based on the inhibition of cytochrome P450 and P-Glycoprotein activities. Front. Pharmacol..

[7] Pel P., Chae H.S., Nhoek P., Yeo W., Kim Y.M., Chin Y.W. (2017). Lignans from the fruits of *Schisandra chinensis* (Turcz.) Baill inhibit proprotein convertase subtilisin/kexin type 9 expression. Phytochemistry.

[8] Szopa A., Ekiert R., Ekiert H. (2017). Current knowledge of *Schisandra chinensis* (Turcz.) Baill. (Chinese magnolia vine) as a medicinal plant species: A review on the bioactive components, pharmacological properties, analytical and biotechnological studies. Phytochem. Rev..

[9] Wang Y., Zhu J., Du X., Li Y. (2023). Simultaneous extraction and determination of lignans from *Schisandra chinensis* (Turcz.) Baill. via diol-based matrix solid-phase dispersion with high-performance liquid chromatography. Molecules.

[10] Liu J., Xiong R., Liu L., Zhou T.P., Xue J., Sun D., Gao R., Chen S., Xu R., Xing Y. (2025). Insights into angiosperm evolution and lineage-specialized lignan biosynthesis from the early-diverging *Schisandra* genome. Sci. Adv..

[11] Qiu F., Liu H., Duan H., Chen P., Lu S.J., Yang G.Z., Lei X.X. (2018). Isolation, structural elucidation of three new triterpenoids from the stems and leaves of *Schisandra chinensis* (Turcz) Baill. Molecules.

[12] Ferrão L.F.V., Sater H., Lyrene P., Amadeu R.R., Sims C.A., Tieman D.M., Munoz P.R. (2022). Terpene volatiles mediates the chemical basis of blueberry aroma and consumer acceptability. Food Res. Int..

[13] Yan Y., Lu W., Tian T., Shu N., Yang Y., Fan S., Han X., Ge Y., Xu P. (2023). Analysis of volatile components in dried fruits and branch exudates of *Schisandra chinensis* with different fruit colors using GC-IMS technology. Molecules.

[14] Chen X., Zhang Y., Zu Y., Yang L. (2012). Chemical composition and antioxidant activity of the essential oil of *Schisandra chinensis* fruits. Nat. Prod. Res..

[15] Wang X.F., Chen X., Tang Y., Wu J.M., Qin D.L., Yu L., Yu C.L., Zhou X.G., Wu A.G. (2022). The therapeutic potential of plant polysaccharides in metabolic diseases. Pharmaceuticals.

[16] Xiao Q., Mu X., Liu J., Li B., Liu H., Zhang B., Xiao P. (2022). Plant metabolomics: A new strategy and tool for quality evaluation of Chinese medicinal materials. Chin. Med..

[17] Tsugawa H., Rai A., Saito K., Nakabayashi R. (2021). Metabolomics and complementary techniques to investigate the plant phytochemical cosmos. Nat. Prod. Rep..

[18] Halket J.M., Waterman D., Przyborowska A.M., Patel R.K., Fraser P.D., Bramley P.M. (2005). Chemical derivatization and mass spectral libraries in metabolic profiling by GC/MS and LC/MS/MS. J. Exp. Bot..

[19] Zeki Ö.C., Eylem C.C., Reçber T., Kır S., Nemutlu E. (2020). Integration of GC-MS and LC-MS for untargeted metabolomics profiling. J. Pharm. Biomed. Anal..

[20] Liu J., Mu X., Liang J., Zhang J., Qiang T., Li H., Li B., Liu H., Zhang B. (2022). Metabolic profiling on the analysis of different parts of *Schisandra chinensis* based on UPLC-QTOF-MS with comparative bioactivity assays. Front. Plant Sci..

[21] Sun B., Yan Y., Ma M., Wen J., He Y., Sun Y., Yuan P., Xu P., Yang Y., Zhao Z. (2024). Based on HPLC and HS-GC-IMS techniques, the changes in the internal chemical components of *Schisandra chinensis* (Turcz.) Baill. fruit at different harvesting periods were analyzed. Molecules.

[22] Ma R., Xiao Y., Lv Z., Tan H., Chen R., Li Q., Chen J., Wang Y., Yin J., Zhang L. (2017). AP2/ERF transcription factor, *Ii049*, positively regulates lignan biosynthesis in *Isatis indigotica* through activating salicylic acid signaling and lignan/lignin pathway genes. Front. Plant Sci..

[23] Samari E., Sharifi M., Karimi F., Sajedi R.H., Sagharyan M. (2025). Regulation of lignan biosynthesis through signaling pathways mediated by H_2_O_2_ in *Linum album* cell culture in response to exogenous putrescine. Sci. Rep..

[24] Xiao Y., Feng J., Li Q., Zhou Y., Bu Q., Zhou J., Tan H., Yang Y., Zhang L., Chen W. (2020). *IiWRKY34* positively regulates yield, lignan biosynthesis and stress tolerance in *Isatis indigotica*. Acta Pharm. Sin. B.

[25] Fakhari S., Sharifi M., De Michele R., Ghanati F., Safaie N., Sadeghnezhad E. (2019). Hydrogen sulfide directs metabolic flux towards the lignan biosynthesis in *Linum album* hairy roots. Plant Physiol. Biochem..

[26] Markulin L., Drouet S., Corbin C., Decourtil C., Garros L., Renouard S., Lopez T., Mongelard G., Gutierrez L., Auguin D. (2019). The control exerted by ABA on lignan biosynthesis in flax (*Linum usitatissimum* L.) is modulated by a Ca^2+^ signal transduction involving the calmodulin-like LuCML15b. J. Plant Physiol..

[27] Hong C.P., Kim C.K., Lee D.J., Jeong H.J., Lee Y., Park S.G., Kim H.J., Kang J.N., Ryu H., Kwon S.J. (2022). Long-read transcriptome sequencing provides insight into lignan biosynthesis during fruit development in *Schisandra chinensis*. BMC Genom..

[28] Lv X., Li L., Lu X., Wang W., Sun J., Liu Y., Mu J., Ma Q., Wang J. (2022). Effects of organic acids on color intensification, thermodynamics, and copigmentation interactions with anthocyanins. Food Chem..

[29] Li H., Fan C., Liu J., Wang B., Li H. (2022). Integration of full-length transcriptomes and anthocyanin metabolite analysis for understanding fruit coloration mechanism in *Schisandra chinensis*. Physiol. Mol. Biol. Plants.

[30] Hu G., Qi Z., Wang A., Jia J. (2020). Effects of Deacidification on Composition of *Schisandra chinensis* Ethanolic Extract and Studies on Acute Toxicity in Mice. Molecules.

[31] Yang S., Shan L., Luo H., Sheng X., Du J., Li Y. (2017). Rapid classification and identification of chemical components of *Schisandra Chinensis* by UPLC-Q-TOF/MS combined with data post-processing. Molecules.

[32] Yan Y., Sun B., Wang M., Wang Y., Yang Y., Zhang B., Sun Y., Yuan P., Wen J., He Y. (2024). Utilizing headspace-gas chromatography-ion mobility spectroscopy technology to establish the volatile chemical component fingerprint profiles of *Schisandra chinensis* processed by different preparation methods and to perform differential analysis of their components. Molecules.

[33] Hazrati S., Mousavi Z., Nicola S. (2024). Harvest time optimization for medicinal and aromatic plant secondary metabolites. Plant Physiol. Biochem..

[34] Xing Z., Bi G., Li T., Zhang Q., Knight P.R. (2024). Effect of harvest time on growth and bioactive compounds in *Salvia miltiorrhiza*. Plants.

[35] Yang P., Tian D., Han X.Y., Zou Q.J., Ma L.J., Wei M., Yu M., Zou Z.M. (2024). Optimal harvest period and quality control markers of cultivated Flos Chrysanthemi Indici using untargeted/targeted metabolomics, chemometric analysis and in vivo study. J. Ethnopharmacol..

[36] Chen J., Guo L., Yang G., Yang A., Zheng Y., Wang L. (2022). Metabolomic profiling of developing perilla leaves reveals the best harvest time. Front. Plant Sci..

[37] Hao J., Wang Z., Jia Y., Sun L., Fu Z., Zhao M., Li Y., Yuan N., Cong B., Zhao L. (2023). Optimization of ultrasonic-assisted extraction of flavonoids from *Lactuca indica* L. cv. Mengzao and their antioxidant properties. Front. Nutr..

[38] Ma B., Ma J., Li B., Tao Q., Gan J., Yan Z. (2021). Effects of different harvesting times and processing methods on the quality of cultivated *Fritillaria cirrhosa* D. Don. Food Sci. Nutr..

[39] Navarro-Reig M., Jaumot J., García-Reiriz A., Tauler R. (2015). Evaluation of changes induced in rice metabolome by Cd and Cu exposure using LC-MS with XCMS and MCR-ALS data analysis strategies. Anal. Bioanal. Chem..

[40] Ogata H., Goto S., Sato K., Fujibuchi W., Bono H., Kanehisa M. (1999). KEGG: Kyoto Encyclopedia of Genes and Genomes. Nucleic Acids Res..

[41] Thévenot E.A., Roux A., Xu Y., Ezan E., Junot C. (2015). Analysis of the human adult urinary metabolome variations with age, body mass index, and gender by implementing a comprehensive workflow for univariate and OPLS statistical analyses. J. Proteome Res..

[42] Xia J., Wishart D.S. (2011). Web-based inference of biological patterns, functions and pathways from metabolomic data using MetaboAnalyst. Nat. Protoc..

[43] Xiao G., Hu Z., Jia C., Yang M., Li D., Xu A., Jiang J., Chen Z., Li Y., Li S. (2023). Deciphering the mechanisms of Yinlan Tiaozhi capsule in treating hyperlipidemia by combining network pharmacology, molecular docking and experimental verification. Sci. Rep..

